# The impact of multi-level interventions on the second-wave SARS-CoV-2 transmission in China

**DOI:** 10.1371/journal.pone.0274590

**Published:** 2022-09-16

**Authors:** Yuanchen He, Yinzi Chen, Lin Yang, Ying Zhou, Run Ye, Xiling Wang

**Affiliations:** 1 School of Public Health, Fudan University, Key Laboratory of Public Health Safety, Ministry of Education, Shanghai, China; 2 School of Nursing, Hong Kong Polytechnic University, Hong Kong, China; 3 School of Public Health, Shenzhen University, Health Science Center, Shenzhen, China; 4 Department of Tropical Diseases, Navy Medical University, Shanghai, China; 5 Shanghai Key Laboratory of Meteorology and Health, Shanghai, China; The Chinese University of Hong Kong, HONG KONG

## Abstract

**Background:**

A re-emergence of COVID-19 occurred in the northeast of China in early 2021. Different levels of non-pharmaceutical interventions, from mass testing to city-level lockdown, were implemented to contain the transmission of SARS-CoV-2. Our study is aimed to evaluate the impact of multi-level control measures on the second-wave SARS-CoV-2 transmission in the most affected cities in China.

**Methods:**

Five cities with over 100 reported COVID-19 cases within one month from Dec 2020 to Feb 2021 were included in our analysis. We fitted the exponential growth model to estimate basic reproduction number (*R*_*0*_), and used a Bayesian approach to assess the dynamics of the time-varying reproduction number (*R*_*t*_). We fitted linear regression lines on *R*_*t*_ estimates for comparing the decline rates of *R*_*t*_ across cities, and the slopes were tested by analysis of covariance. The effect of non-pharmaceutical interventions (NPIs) was quantified by relative *R*_*t*_ reduction and statistically compared by analysis of variance.

**Results:**

A total of 2,609 COVID-19 cases were analyzed in this study. We estimated that *R*_*0*_ all exceeded 1, with the highest value of 3.63 (1.36, 8.53) in Haerbin and the lowest value of 2.45 (1.44, 3.98) in Shijiazhuang. Downward trends of *R*_*t*_ were found in all cities, and the starting time of *R*_*t*_ < 1 was around the 12th day of the first local COVID-19 cases. Statistical tests on regression slopes of *R*_*t*_ and effect of NPIs both showed no significant difference across five cities (*P* = 0.126 and 0.157).

**Conclusion:**

Timely implemented NPIs could control the transmission of SARS-CoV-2 with low-intensity measures for places where population immunity has not been established.

## Introduction

The emergence of coronavirus disease 2019 (COVID-19), which was first detected in December 2019 in Wuhan, China, has resulted in over 188 million cases and 4 million deaths around the world as of 16 July 2021 [1]. The first-wave of epidemic has been contained effectively by a series of stringent public health interventions in mainland China, especially the decisive city-level lockdown [2, 3]. The absence of herd immunity [4–6] and emerging SARS-CoV-2 variants still posed an upcoming threat of re-emergence to the whole society. After relaxing the interventions and resuming a normal life, the northeast cities of China experienced the second-wave of COVID-19 epidemic from Dec 2020 to Feb 2021. The first confirmed local case was reported on 2 Jan 2021 in Shijiazhuang city, Hebei province, and was likely originated from Russian strains suggested by genomic sequencing [7]. The epidemic spread rapidly to over 10 neighboring cities in Heilongjiang, Jilin and Liaoning provinces, within only 10 days.

**Fig 1:**
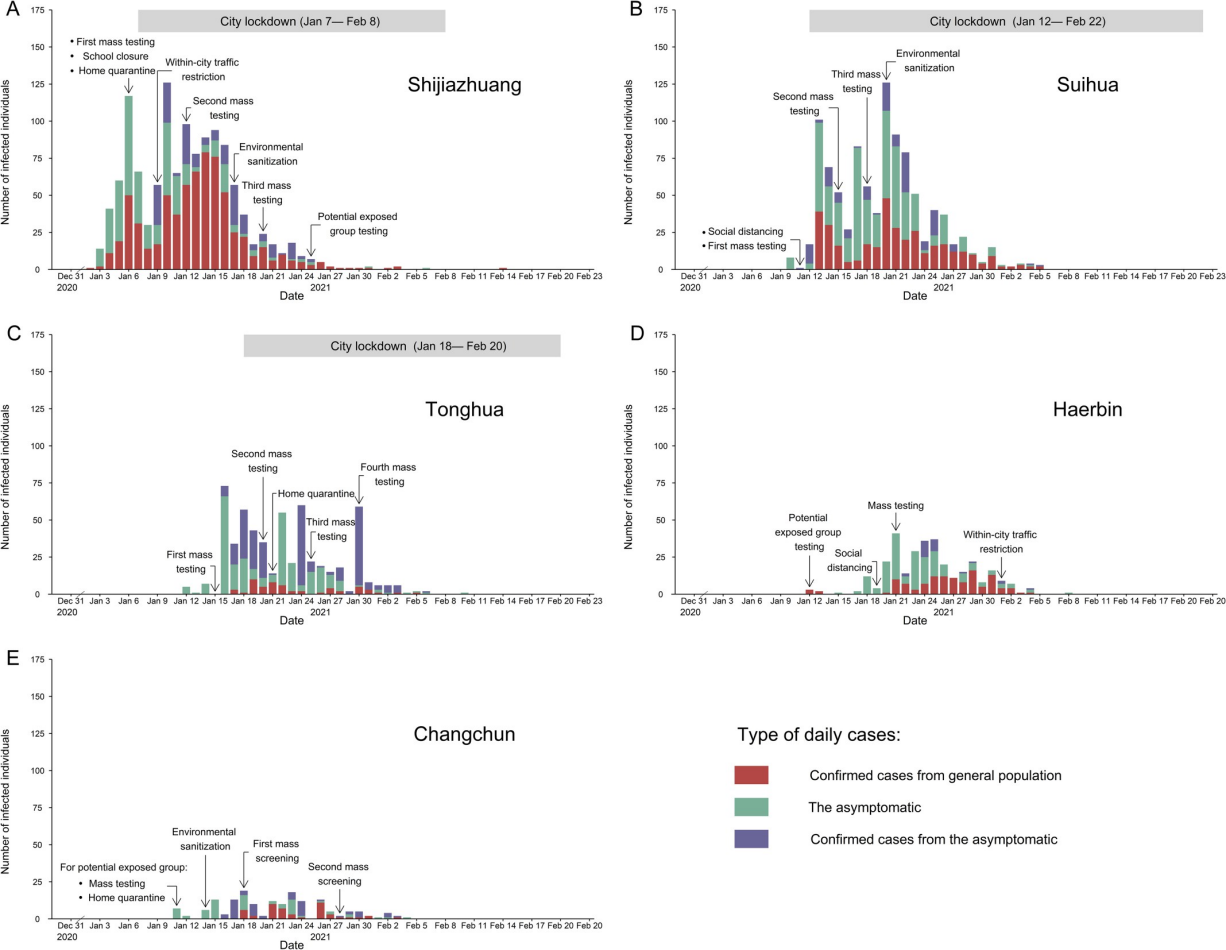
Epidemic curves and non-pharmaceutical interventions implemented in five worst-hit cities in China, Jan-Feb 2021. (A)-(E) The daily number of COVID-19 infections and the timings of specific non-pharmaceutical interventions in Shijiazhuang, Suihua, Tonghua, Haerbin and Changchun cities.

As the COVID-19 vaccine campaign was not launched in mainland China until early 2021, non-pharmaceutical interventions (NPIs) remained the main way to control the second-wave of COVID-19. The effects of NPIs on controlling COVID-19 transmission have been widely confirmed in the first- and second-wave [8–12]. A study from Italy by Manica et al. [13] has estimated that the three-tiered restriction system, which was taken by Italian government to counter the national second-wave, effectively lowered the reproduction number below 1 in 85 out of 107 provinces. While some researchers believed that without strengthening interventions, it would be exceedingly difficult to contain a COVID-19 resurgence [14, 15] even as vaccination is coming into effect. China has adopted different levels of control measures including mass testing, social distancing and lockdown in cities since 6 Jan 2021 (Fig 1), but there is limited literature so far to investigate whether early implementing NPIs could avoid strong-intensity control measures. In this study, we aimed to assess the impact of multi-level interventions on SARS-CoV-2 transmission in the worst-hit cities during the second wave of COVID-19 in China.

## Materials and methods

### City selection and case definition

We restricted analyses to prefecture-level cities that reported the total number of confirmed COVID-19 cases and asymptomatic SARS-CoV-2 infected individuals over 100 within a consecutive month during Dec 2020 to Feb 2021. The definition of symptomatic and asymptomatic COVID-19 cases (details in S1 File) followed the Protocol on Prevention and Control of Novel Coronavirus Pneumonia (7th Edition) [16] issued on Sept 11, 2020 by Chinese Center for Disease Control and Prevention (China CDC). All SARS-CoV-2 infected individuals would be quarantined and treated by municipal designated hospitals.

### Data collection

The daily cumulative number of confirmed COVID-19 cases and asymptomatic cases were extracted from the official websites of national, provincial, and municipal health commissions. Individual information on COVID-19 cases, including diagnosis date, type of SARS-CoV-2 infected individuals (symptomatic or asymptomatic) and source of infection (imported from other cities or local transmission) was collected from open access municipal official press release platforms.

The NPIs taken by local governments were collected from two publicly official sources: the press conferences on the novel coronavirus and authorized governmental news media. Following the protocol [16] from China CDC and guidelines from WHO [17], we summarized 8 types of main interventions (as defined in S1 File), including testing (mass/for the potential exposed), school closure, home quarantine, within-city traffic restriction, environmental sanitization, social distancing and lockdown. Further, we reclassified the interventions into three levels according to their influence on transmission rate. Based on previous studies [18, 19], we classified mass testing and potential exposed group testing as “Mild” NPIs, city lockdown as “Strong” NPIs and the rest of interventions as “Moderate” NPIs.

### Statistical analysis

Considering that less than 0.02‰ of the people in the selected provinces had been confirmed as COVID-19 cases before second-wave (the Note 3 of S1 File), basic reproduction numbers of selected cities, denoted by *R*_*0*_, were estimated through the exponential growth rate *r* as in Formulation (1).

$$R_{0}=\frac{1}{M(-r)}$$ (1)

where *M* is the moment generating function of the discretized generation time distribution [20]. The appropriate expression of *M* is determined by the shape of generation time distribution. In the main analysis, the generation time (i.e., the lag between infection of the primary case and of their secondary cases) was assumed to follow a gamma distribution with a mean (SD) of 7.5(3.4) days [21]. Alternative scenarios about the values of the serial interval were explored in sensitivity analyses, one with mean (SD) of 5.7(1.8) days [22] and the other with mean (SD) of 5.0(1.7) days [23]. Simple moving average (n = 7) was used to acquire smoother epidemic curves and fit exponential growth. The time period used to fit the exponential growth was selected based on the highest R-squared values.

Time-varying reproduction number (*R*_*t*_) was estimated by the Cori method [24] using the daily number of confirmed and asymptomatic cases by their diagnosis date (the Note 4 of S1 File). Using the infection-to-diagnosis delay distribution, the time series of daily number of diagnosis was deconvoluted to the time series of daily number of new infections [25, 26]. This estimation method specifically distinguishes between local cases and imported cases [27] to better estimate community transmission. To fulfil the criteria for when to start *R*_*t*_ estimation required by the method (listed in the Note 5 and 6 of S1 File), we started the calculation from the 7th day of the first local cases (including confirmed and asymptomatic cases). We assumed the relative infectiousness of the asymptomatic and pre-symptomatic were 50% and 100% of the symptomatic transmission, and set the time window τ to 7 days. Simple linear regression lines were fitted to daily *R*_*t*_ values from the start of estimation to the first day of *R*_*t*_ < 1 in each city, and analysis of covariance (ANCOVA) was used to test the statistical difference of regression slopes of *R*_*t*_. To evaluate the impact of NPIs on controlling COVID-19 transmission, we also used the following Formulation (2) to quantify the relative changes of *R*_*t*_ [28].

$$\varepsilon =\frac{R_{t_{0}}-1}{t_{1}-t_{0}}$$ (2)

where ε is the controlling effect indicator, t_0_ and t_1_ is the time when *R*_*t*_ estimation onset and the minimum time when the mean *R*_*t*_ < 1. $R_{t_{0}}$ represents the corresponding *R*_*t*_ value at time t_0_. The indicators were statistically compared by analysis of variance (ANOVA). For comparing the timeliness of taking measures across the cities, we also calculated the delay from the first case confirmation to the first intervention implementation and the cumulative cases when imposing the first intervention respectively. Statistical analyses and visualization were all performed using the R software (version 4.1.0), the ANCOVA and ANOVA tests were implemented via *stats* and *ggpmisc* packages. Statistical significance was considered to be *P* < 0.05.

## Results

### City description

During Dec 2020 to Feb 2021, there were 5 cities reporting over 100 COVID-19 cases within a month (Fig 1). For Shijiazhuang, Suihua and Tonghua cities, the total numbers of confirmed and asymptomatic cases were 1043, 844 and 320 respectively, followed by 291 and 111 in Haerbin and Changchun cities. The second-wave of COVID-19 was first detected in Shijiazhuang city, Hebei Province on 2 Jan 2021 and then spread to the cities of neighboring provinces (e.g., Jilin and Heilongjiang Provinces). Other four cities (Suihua, Tonghua, Haerbin and Changchun city) began reporting local cases on Jan 10, 12, 15 and 11. We included 1351 asymptomatic cases in the analyses, among whom 658 (48.7%) were re-diagnosed as confirmed COVID-19 cases later. Shijiazhuang city had the most SARS-CoV-2 infected individuals (n = 1043) and the longest epidemic period (44 days) in the epidemic.

### Epidemic curves and NPIs

Starting from the end of 2020, the second-wave of COVID-19 grew rapidly in the northeast of China, especially in Shijiazhuang, Suihua and Tonghua cities. Haerbin and Changchun cities implemented NPIs on the same day of reporting the first case, while the other three cities delayed actions by 1–4 days as shown in Table 1. The cumulative cases were 233, 8, 13, 3 and 7 respectively in the cities when the first intervention was implemented. In the epidemic, the municipal CDC implemented mass testing for case investigation and contact tracing. Each city implemented its most stringent control measures within a week from the start of local epidemic.

**Table 1:** The description of COVID-19 epidemic before imposing NPIs in five worst-hit cities in China, Jan-Feb 2021.

City	1st case reported	1st intervention imposed	Lag time^a^ (days)	Cumulative cases^b^
Shijiazhuang	Jan 02	Jan 06	4	233
Suihua	Jan 10	Jan 11	1	8
Tonghua	Jan 12	Jan 15	3	13
Haerbin	Jan 12	Jan 12	0	3
Changchun	Jan 11	Jan 11	0	7

^a^: The time from the date that reported the first case to the date that imposed the first intervention.

^b^: The number of cumulative cases was counted until the date that imposed the first intervention.

Notably, the three worst-hit cities, Shijiazhuang, Suihua and Tonghua, took “Strong” measures (city lockdown) over a month (implemented in Jan 7- Feb 8, Jan 12- Feb 22 and Jan 18- Feb 20 respectively) to contain the epidemic. The cumulative cases in these three cities were 299, 12 and 122 respectively on the first day of city lockdown. These three cities kept multiple rounds of mass testing during the lockdown period, Shijiazhuang also implemented environmental sanitization, within-city traffic restriction and potential exposed group testing. In addition, Suihua city adopted environmental sanitization on Jan 20 as Shijiazhuang did on Jan 17.

Haerbin and Changchun cities only adopted “Mild” and “Moderate” control measures instead. The highest city-level measures were initiated when local cases accumulated to 14 and 8 respectively, which specifically referred to within-city traffic restriction and environmental sanitization. Besides, the testing strategies in the two cities were both transferred from potential exposed group testing to mass testing during the “Moderate” period.

### Basic reproduction number (*R*_*0*_) and time-varying reproduction number (*R*_*t*_)

The start of the best fitted time period lagged 4–9 days behind the start of epidemic time (curves after moving average were shown in S1 Fig of S1 File). All cities’ *R*_*0*_ values were well above 1 (Table 2), with the highest in Haerbin (3.63, 95% CI: 1.36–8.53) and the lowest in Shijiazhuang (2.45, 95% CI: 1.44–3.98). Different generation time distributions showed similar results of *R*_*0*_ with main analysis, which validated robustness of *R*_*0*_ (in the S2 Fig of S1 File).

**Table 2:** Basic reproduction number (*R*_*0*_) estimated by the exponential growth method.

City	Epidemic time period	Best fitted time period	R_0_ (95% CI)	${R^{2}}^{a}$
Shijiazhuang	01.02–02.14	01.07–01.12	2.45 (1.44, 3.98)	0.966
Suihua	01.10–02.05	01.14–01.19	3.04 (1.61, 5.43)	0.985
Tonghua	01.12–02.10	01.16–01.22	3.26 (1.46, 6.72)	0.969
Haerbin	01.12–02.08	01.21–01.26	3.63 (1.36, 8.53)	0.988
Changchun	01.11–02.04	01.15–01.24	2.72 (1.18, 5.82)	0.961

^a^: R-squared values, which was used to select the best fitted time period.

**Fig 2:**
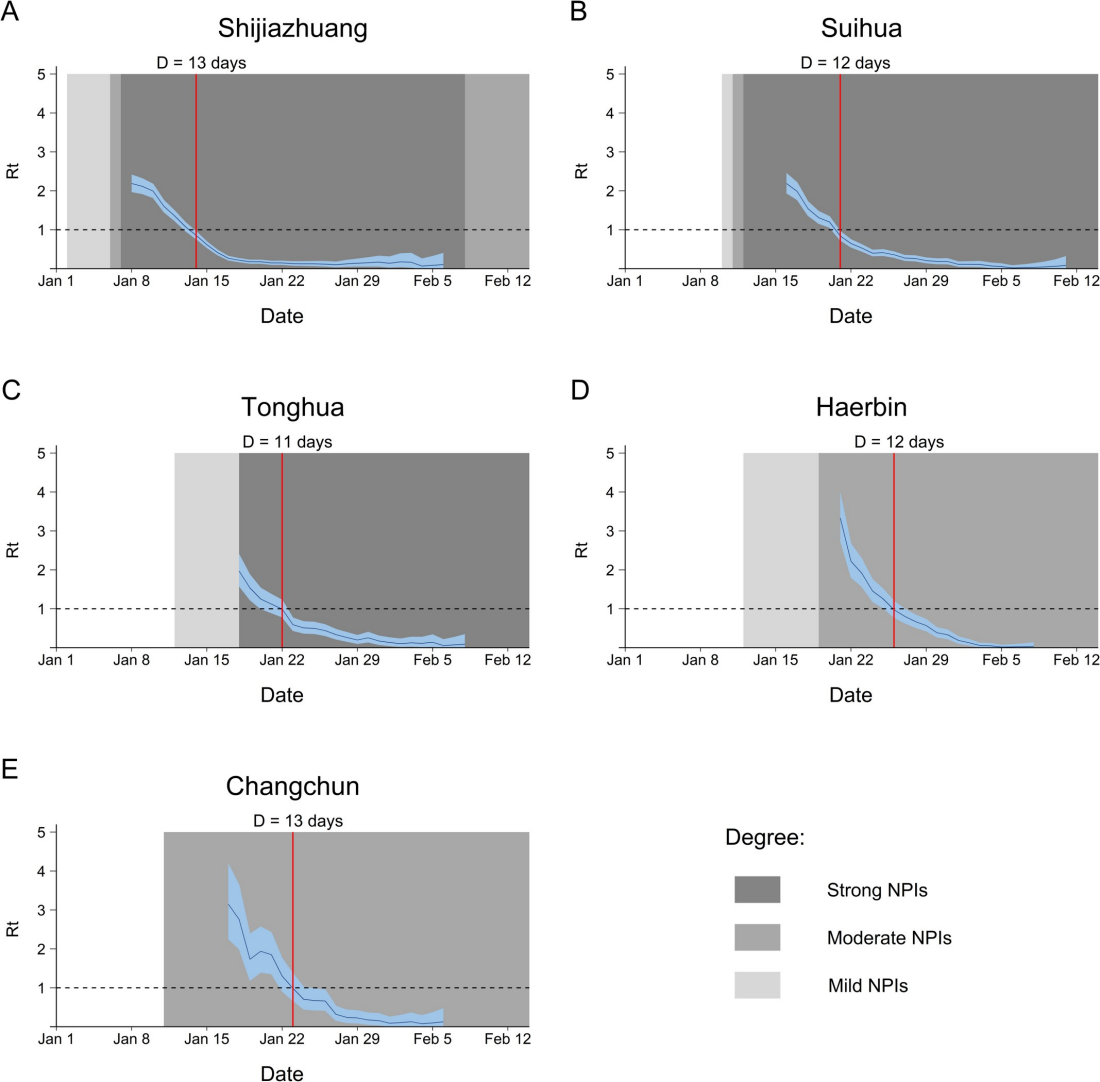
Temporal dynamics of SARS-CoV-2 transmission in five worst-hit cities in China, Jan-Feb 2021. The light, medium and dark grey regions show periods of mild, moderate and strong NPIs respectively. The red line represents the first day of *R*_*t*_ < 1 in each city. D represents the days from the initial identification of local cases to *R*_*t*_ below the threshold. (A) Estimated *R*_*t*_ values from Jan 8 to Feb 6 in Shijiazhuang city. (B) From Jan 16 to Feb 11 in Suihua city. (C) From Jan 18 to Feb 8 in Tonghua city. (D) From Jan 21 to Feb 8 in Haerbin city. (E) From Jan 17 to Feb 6 in Changchun city. *R*_*t*_ = time-varying reproduction number.

**Fig 3:**
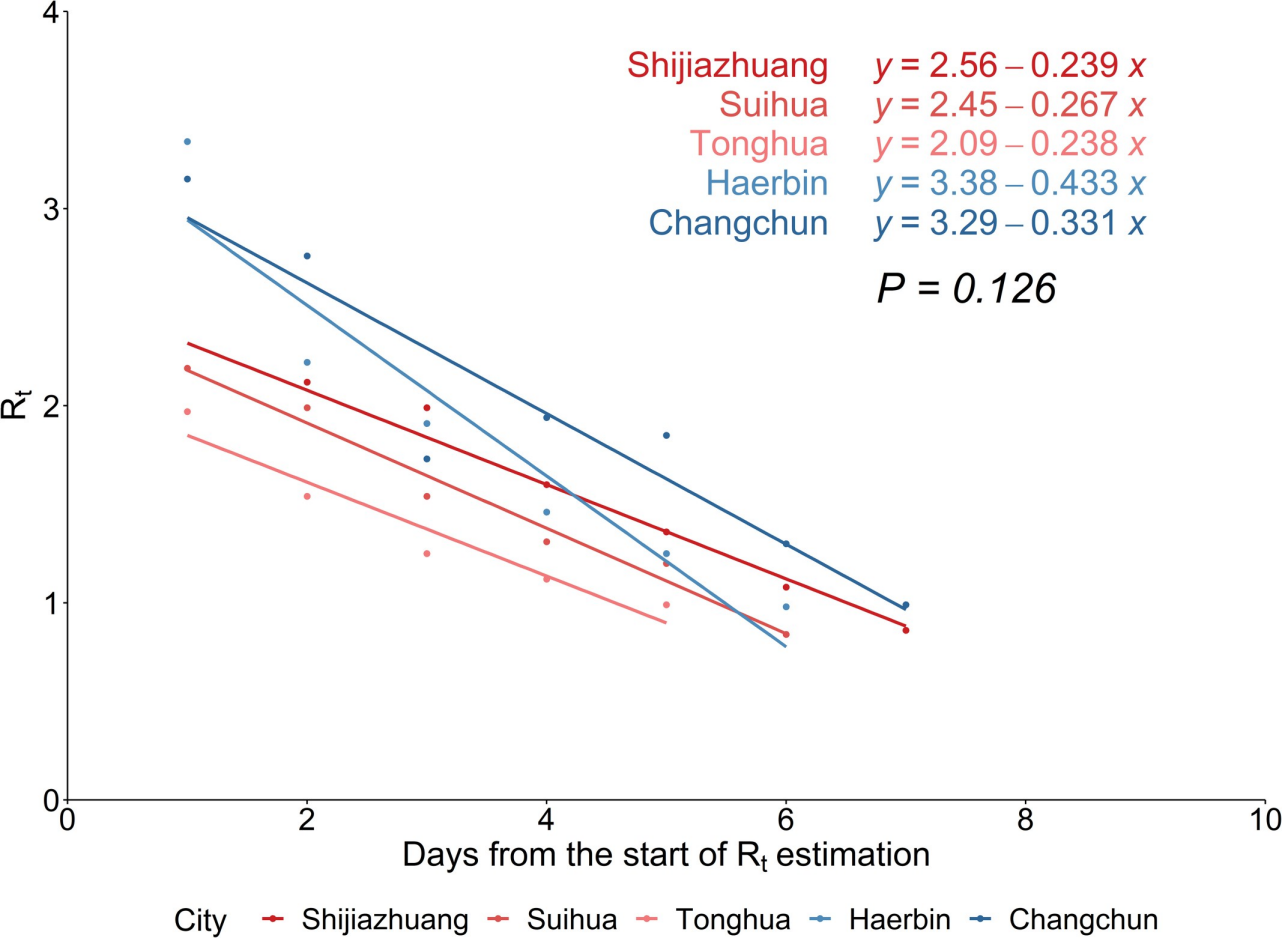
The scatter plots and fitted regression lines of *R*_*t*_ estimations in five worst-hit cities in China. The “red” lines represent the cities with “Strong” level NPIs, the “blue” lines represent the cities without “Strong” level NPIs. The equations of linear regression (top right) were fitted from the *R*_*t*_ values from the start of calculation to the first day of *R*_*t*_ < 1 in each city. The *P* value below the equations were the *p*-value for the hypothesis test based on the analysis of covariance (ANCOVA) evaluating whether the decline in transmission rate was different across cities. *R*_*t*_ = time-varying reproduction number.

The *R*_*t*_ values of Shijiazhuang, Suihua, Tonghua and Haerbin city showed a continuous downward trend (Fig 2), while those of Changchun city showed a small rise on January 20 followed by a steady decline. The starting time of *R*_*t*_ < 1 was not the same calendar time between these 5 cities, but around the day 11 to 13 of reporting local COVID-19 cases. The daily estimated values of *R*_*t*_ in all cities are shown in S1 File. When different levels of interventions were taken at the same time, we only retain the highest level of NPIs in each city for visualization (Fig 2). The results revealed that the highest level of NPIs had been implemented before or exactly the day we began to estimate effective reproduction number, and *R*_*t*_ kept falling since the implementation. Fitting the regression lines with *R*_*t*_ of each city, the slopes were not statistically different (*P* = 0.126, Fig 3). Comparing the effectiveness of NPIs as shown in Table 3, Haerbin city had the highest effect among five cities though without city lockdown, while Shijiazhuang city had the lowest effect with the most kinds of NPIs implemented. The effectiveness of NPIs were not significantly different as the P value was above 0.05.

**Table 3:** The effectiveness of NPIs in five worst-hit cities in China.

City	$R_{t_{0}}$	(t_1_ –t_0_)/days	ε (95% CI)	*P* value
Shijiazhuang	2.19 (1.98, 2.41)	6	0.198 (0.163, 0.235)	0.157
Suihua	2.19 (1.94, 2.45)	5	0.238 (0.188, 0.290)	
Tonghua	1.97 (1.59, 2.40)	4	0.243 (0.148, 0.350)	
Haerbin	3.34 (2.76, 3.97)	5	0.468 (0.352, 0.594)	
Changchun	3.15 (2.26, 4.18)	6	0.358 (0.210, 0.530)	

## Discussion

Here we retrospectively assessed the transmission potentials and epidemic evolution of COVID-19 using *R*_*t*_ in northeast of mainland China, the worst affected area in the second-wave epidemic. Three of the cities included in the study adopted “Strong” level NPIs, while the other two only implemented “Mild” and “Moderate” level NPIs. The *R*_*t*_ in all five cities fell below 1 within 2 weeks from the first case confirmation. The decline rate of *R*_*t*_ and effectiveness of NPIs were not significantly different across cities.

The estimates of *R*_*0*_, ranging from 2 to 4, showed no significant difference among selected cities. Reviewing the first wave, a narrow range of *R*_*0*_, between two and three, were frequently estimated from data on confirmed cases [29–31]. Specifically in some studies that located in Wuhan, China [21, 32–35], range of *R*_*0*_ estimates reported from at least 2.20 to 2.71. The contact patterns after the first wave couldn’t be comparable with the pre-pandemic level, even with a few restriction policies. The mean number of contacts only reached about 50% or less of the pre-pandemic level [36–38]. Considering the transmissibility of SARS-CoV-2, which usually measured by *R*_*0*_, was significantly influenced by population contact level, *R*_*0*_ in the first- and second- waves should be compared with caution and required more information.

The rapid decline of *R*_*t*_ suggests the effectiveness of non-pharmaceutical interventions implemented in five cities. Owing to stringent NPIs implemented in the early phase, *R*_*t*_ has been below the epidemiological threshold as of Jan 26, 2021 in all cities. The comparable *R*_*t*_ slopes suggested that with only “Mild” and “Moderate” measures, Haerbin and Changchun cities still successfully drove *R*_*t*_ below 1 as in other three cities. The prominent effectiveness enlightened that if NPIs are taken as soon as possible, as Haerbin and Changchun city began to implement city-wide measures when there were few sporadic cases, the highest level of interventions (i.e., city lockdown) may not be needed, thus reducing the impact on socioeconomics. The heterogeneity of sociodemographic and geographic characteristics in five cities may confound the association between *R*_*t*_ and effectiveness of NPIs, but the confounding effect might be minor. Geographically, the five cities are in the Hebei, Heilongjiang and Jilin provinces, concentrated in northeast China. For the age structure, the proportion of population aged 0–14, 15–59, and 60+ ranged from 10–19%, 62–67% and 18–25%, respectively [39–42]. Economically, these cities mainly develop heavy industry with the per capita GRP in 2020 being 52.961, 30.257, 34.338, 51.597 and 77.634 thousand respectively in Shijiazhuang, Suihua, Tonghua, Haerbin and Changchun cities, reported by the official Statistical Yearbook [43–45]. In the analysis, we classified interventions that besides testing and lockdown policies, rest of the interventions were all at the “Moderate” level. The individual effects of these interventions were hard to quantify in reality partly because of close intervals in implementation [19] and partly because of the limited impact on *R*_*t*_ with a single control measure [46]. The concurrent interventions might obscure the association between *R*_*t*_ and the impact of NPI at a specific level, which required further exploration.

A world research found even with concurrent lockdown policies, school closure and social distancing still worked on reducing COVID-19 infections. But the within-city traffic restriction had no such effect [47]. Another systematic review also found that no evidence on the effectiveness of public transport closure [48]. This counterintuitive result might be due to the interventions imposed before the traffic restriction had reduced human mobility already, as shown in Shijiazhuang and Haerbin cities. These research enlightened that effects of interventions in the “Moderate” level might vary and need more detailed exploration.

The study presented here is subject to several limitations. First, the study focused primarily on population-based rather than individual-based interventions, but targeted test, trace and isolation strategy might be also effective to prevent further COVID-19 transmission [49]. On the other hand, the success of individual-based interventions relies highly on the response speed of disease surveillance system in the early stages [23, 50]. As this was a pronounced challenge for places where community transmission had occurred, Shijiazhuang, Suihua and Tonghua implemented mass testing as the first intervention to quickly identify exposed population. Second, we extracted the parameters of generation time distribution from published literatures. As the virus mutating, the epidemiological characteristics of first-wave might not be suitable for this epidemic in mainland China. But sensitivity analyses of R_0_ by changing the generation time reveled the robustness of our estimates. Third, to our knowledge, an asymptomatic individual infected 102 new cases across three cities through crowded indoor lectures in this epidemic. Considering the source of superspreading events was from Suihua city and the efficacy of contact tracing inevitably wouldn’t reach 100% [51], even only lagging for 1 day to impose NPIs, Suihua still take city lockdown to contain the epidemic. The key point is that reproduction number only represents average number of secondary cases in a region [52]. This average ignores the 80/20 rule [53], which also means that superspreading events are not detected by *R*_*0*_ or *R*_*t*_ and hard to capture on a population level.

## Conclusion

In conclusion, our results provided an overview and quantify the effects of multi-level interventions of second-wave COVID-19 epidemic in mainland China. The findings suggested a fast downward trend of the COVID-19 outbreak in the northeast provinces, indicating that non-pharmaceutical interventions still played an integral part in stopping the epidemic as in the first-wave, and decisive enforcement might even have dramatic effects. Our study indicated that timely implemented non-pharmaceutical interventions could control the transmission of SARS-CoV-2 with low-intensity measures for places where population immunity have not been established.

## Supporting information

S1 File Appendix. (PDF)

S2 File The daily number of reported cases in five cities. (XLSX)
