# Supplementary material for: The impact of multi-level interventions on the second-wave SARS-CoV-2 transmission in China
Source: PLoS One. 2022 Sep 16;17(9):e0274590. doi: 10.1371/journal.pone.0274590 (PMC9481005; doi:10.1371/journal.pone.0274590)
Supplement: S1 File — (PDF) [file pone.0274590.s001.pdf]

The impact of multi-level interventions on the second-wave SARS-CoV-2 transmission in China

Supplementary material

Yuanchen He, BSc[1], Yinzi Chen, BSc[1], Lin Yang, PhD[2], Ying Zhou, PhD[3], Run Ye, PhD[4], Xiling Wang, PhD[1,5*]

Author affiliations:

1. School of Public Health, Fudan University, Key Laboratory of Public Health Safety, Ministry of Education, Xuhui District, Shanghai 200231, China

2. School of Nursing, Hong Kong Polytechnic University, Hong Kong, China

3. School of Public Health, Shenzhen University, Health Science Center, Shenzhen 518000, China

4. Department of Tropical Diseases, Navy Medical University, Shanghai 200433, China

5. Shanghai Key Laboratory of Meteorology and Health, Shanghai, China

* These authors are joint senior authors and contributed equally to this work.

Contents

Supplementary Note 1. Definitions of cases of SARS-CoV-2 infection and non-pharmaceutical interventions 3

Supplementary Note 2. Cumulative confirmed COVID-19 cases before Jan 2, 2021 in Hebei, Jilin and Heilongjiang provinces........ 7

Supplementary Note 3. Estimation of time-varying reproduction number 8

Supplementary Note 4. Criteria for choosing time window ($\boldsymbol{\tau}$) and the start time of R_t calculation........10

Supplementary Note 5. Daily values of effective reproduction number (R_t) 12

Figure S1. Daily new COVID-19 infections and moving average curves at city-level.. 16

Figure S2. Estimates of R_0 with different generation time distributions........17

Supplementary References 18

Supplementary Note 1. Definitions of cases of SARS-CoV-2 infection and non-pharmaceutical interventions

Definitions of cases of SARS-CoV-2 infection

Confirmed COVID-19 cases

A confirmed COVID-19 case was defined as a person who: i) met two or more clinical criteria (fever with or without respiratory symptoms; radiographic characteristics of COVID-19; laboratory evidence of normal or decreased count of leukocytes and/or lymphopenia in the early stage of the disease), ii) had an epidemiological link to a SARS-CoV-2 positive individual or had been to regions reporting SARS-CoV-2 transmission within 14 days before disease onset (including clustering transmission), iii) had a positive real-time RT-PCR result or the virus genetic sequence matching known strains of SARS-CoV-2.

Asymptomatic SARS-CoV-2 infected individuals

An asymptomatic SARS-CoV-2 infected individual was defined as a person whose upper respiratory specimens tested positive in real-time RT-PCR without any identifiable clinical symptoms or any COVID-19-related CT imaging characteristics. When showing COVID-19-related signs or symptoms, the asymptomatic cases would be re-diagnosed as confirmed COVID-19 cases within 24 hours.

Classification of SARS-CoV-2 infected individuals

In the analyses, the SARS-CoV-2 infected individuals were categorized into 3 types

as the asymptomatic, confirmed cases from general population and confirmed cases from the asymptomatic. The asymptomatic referred to those who had been diagnosed and first reported as asymptomatic cases without considering whether they would later become confirmed cases or not. Confirmed cases from general population referred to those who had been diagnosed and first reported as confirmed cases following the case definitions listed above. Confirmed cases from the asymptomatic referred to those who were diagnosed and first reported as asymptomatic cases and re-diagnosed as confirmed cases later, also known as pre-symptomatic cases. The daily incremental number of SARS-CoV-2 infected person were only composed of two parts, the asymptomatic and confirmed cases from general population.

Definitions of non-pharmaceutical interventions

Mass testing

This intervention refers to citywide SARS-CoV-2 testing, the date used for visualization is the date when the testing begins to be enforced in the affected city. To detect the asymptomatic and cut off the transmissions as soon as possible, mass testing is implemented weekly by the municipal Center for Disease Control and Prevention (CDC) in the second wave.

Potential exposed group testing

With the demand of region-specific, multi-level targeted approach to prevent and

control epidemic, this policy refers to testing for close contacts of confirmed cases and the asymptomatic, as well as residents in the same and nearby communities.

School closure

This intervention is mainly aimed at the after-school training institutions, while in the case of Shijiazhuang, it refers to all primary and secondary schools offline education suspend since the risk level of affected region has been raised to “High Risk” level.

Home quarantine

It refers to stay at home and separate from others for “14+7” days at the family unit. During the quarantine time, everyone has to take SARS-CoV-2 tests at day 1,7,13 and 20 for COVID-19 case identification.

Within-city traffic restriction

Different from the lockdown, this intervention specially limits the traffic within the city partly or wholly, including taxi, bus and subway.

Environmental sanitization

To minimize the burden of environment associated infection, the governments organize a thorough terminal disinfection across the entire city.

Social distancing

As one of the common interventions against the spread of the COVID-19 epidemic, this measure includes stopping aggregative activities, closing non-essential public places and restricting all other unnecessary contacts.

Lockdown

The intervention is the strictest COVID-19 epidemic control measures which incorporates implementation of closed-end management, closure of assembly occupancies, ordering people to stay at home. In other words, lockdown encompasses all other interventions mentioned above.

Supplementary Note 2. Cumulative confirmed COVID-19 cases before Jan 2, 2021 in Hebei, Jilin and Heilongjiang provinces

To justify the estimates of R_0 in the main analyses, we collected data on cumulative confirmed cases in Hebei, Jilin and Heilongjiang provinces before the second wave in mainland China (retrospective data at the prefectural-city level were hard to obtain from official public sources). As shown in Table S1, comparing with the permanent residents, the proportions of cumulative confirmed cases were less than 0.02‰ in all provinces.

Table S1. The proportion of cumulative confirmed cases before Jan 2, 2021

Province	Cumulative confirmed cases	Permanent residents*	Proportion (‰)
Hebei	339	74610235	0.005
Jilin	138	24073453	0.006
Heilongjiang	580	31850088	0.018

*: The data were extracted from The Seventh National Census in China[1].

Supplementary Note 3. Estimation of time-varying reproduction number

Following Cori[2], the infectiousness profile was assumed to be independent of calendar time t and COVID-19 transmission was modelled with a Poisson process. Therefore the rate at which someone infected in time step t-s generates new infections in time step t is equal to $R_t w_s$, where R_t is the reproduction number at time t and w_s is the distribution of average infectiousness profile after infection. The number infected at time t followed a Poisson distribution with mean $R_t \sum_{s=1}^{t} I_{t-s} w_s$, and conditional on the previous incidences $I_0, \ldots, I_{t-1}$, the likelihood of the incidence I_t is:

$$P(I_t | I_0, \ldots, I_{t-1}, w, R_t) = \frac{(R_t \Lambda_t)^{I_t} e^{-R_t \Lambda_t}}{I_t!}$$

with $\Lambda_t = \sum_{s=1}^{t} I_{t-s} w_s$. If the transmission rate of COVID-19 assumed to be constant within the time window [t-τ+1, t], which is measured by $R_{t,\tau}$. Conditional on the previous incidences $I_0,\ldots,I_{t-\tau}$, the likelihood of the incidence during this time period, $I_{t-\tau+1},\ldots,I_t$, is:

$$P\left(I_{t-\tau+1}, \ldots, I_t \middle| I_0, \ldots, I_{t-\tau}, w, R_{t,\tau}\right) = \prod_{s=t-\tau+1}^{t} \frac{(R_{t,\tau} \Lambda_s)^{I_s} e^{-R_{t,\tau} \Lambda_s}}{I_s!}$$

Using a Bayesian framework with a gamma distribution with parameters (a, b) for $R_{t,\tau}$, then the posterior distribution of $R_{t,\tau}$ is:

$$P\left(I_{t-\tau+1}, \ldots, I_t, R_{t,\tau} \middle| I_0, \ldots, I_{t-\tau}, w\right) = P\left(I_{t-\tau+1}, \ldots, I_t \middle| I_0, \ldots, I_{t-\tau}, w, R_{t,\tau}\right) P\left(R_{t,\tau}\right)$$

$$= R_{t,\tau}^{a+\sum_{s=t-\tau+1}^{t} I_s - 1} e^{-R_{t,\tau}\left(\sum_{s=t-\tau+1}^{t} \Lambda_s + \frac{1}{b}\right)} \prod_{s=t-\tau+1}^{t} \frac{\Lambda_s^{I_s}}{I_s} \frac{1}{\Gamma(a) b^a}$$

Therefore, the posterior distribution of $R_{t,\tau}$ is a gamma distribution with

parameters $(a + \sum_{s=t-\tau+1}^{t} I_s,\ \frac{1}{\frac{1}{b}+\sum_{s=t-\tau+1}^{t} \Lambda_s})$. In our analyses, we used a gamma prior distribution with a = 5 and b = 5 to estimate $R_{t,\tau}$. The conservative choice of parameters ensured that the prior knowledge had limited distractions on the results[3].

Supplementary Note 4. Criteria for choosing the time window (τ) and the start time of R_t calculation

The estimates of R_t are considered to depend on the choice of time window. A small time window τ leads to a faster detection of changes in COVID-19 transmission rate, while a large value of τ leads to a more smoothing and less noisy estimation.

Cori[2] provides an analytical formulation of the posterior distribution of reproduction number, which links the posterior coefficient of variance (referred to as CV) to the number of incident cases in the time window. Here we chose 0.4 as the aimed posterior CV, the threshold (as advised by Table S2) was 7 local incident cases. To satisfy the threshold, we first selected τ = 4, 5, 6 and 7 days to calculate the numbers of incident cases in all cities. Since in the early and final stages of the epidemic, there were only sporadic local cases, τ = 7 days was regard as a proper choice finally.

Following Cori, the start time of R_t calculation in our model had to fulfil these criteria: (i) Firstly, the reproduction number must be calculated at least after one time window (we set time window τ as 7 days in the analyses), as a result of the estimate can only be obtained at the end of a time window. (ii) Secondly, as discussed in supplementary note 4, to get the aimed posterior CV, each time window must comprise at least 7 local incident cases. (iii) Estimating R_t before one generation of cases may induce the associated bias at early stage of an outbreak,

especially when the major part of incident cases is imported cases. It's better to wait until one average generation time to estimate R_t.

Table S2. The threshold for number of incident cases in each time window

Prior CV	Aimed posterior CV									
	**1**	**0.9**	**0.8**	**0.7**	**0.6**	**0.5**	**0.4**	**0.3**	**0.2**	**0.1**
**1**	0	1	1	2	2	3	6	11	24	99
**2**	1	1	2	2	3	4	6	11	25	100
**5**	1	2	2	3	3	4	7	12	25	100
**10**	1	2	2	3	3	4	7	12	25	100
**10000**	1	2	2	3	3	4	7	12	25	100

Supplementary Note 5. Daily values of effective reproduction number (R_t)

The second wave of COVID-19 in mainland China didn't emerge in all cities at the same calendar time. The epidemic first reported in Shijiazhuang city, then spread to other 4 cities. We chose the first day reporting local cases (including confirmed and asymptomatic cases) as day 1. For Shijiazhuang, Suihua, Tonghua, Haerbin and Changchun city, the date of day 1 is January 2, 10, 12, 15 and 11, 2021, respectively. Following Cori method[2], we began to estimate R_t in each city from the day 7 with a sliding time window of 7 days. The results of daily R_t values are shown in **Table S3-S7**.

Table S3. Estimates of the effective reproduction number (R_t) in Shijiazhuang city

Date	Mean (95% Credible interval)
**Jan 8**	2.19 (1.98, 2.41)
**Jan 9**	2.12 (1.92, 2.31)
**Jan 10**	1.99 (1.82, 2.17)
**Jan 11**	1.60 (1.46, 1.76)
**Jan 12**	1.36 (1.23, 1.49)
**Jan 13**	1.08 (0.97, 1.19)
**Jan 14**	0.86 (0.77, 0.96)
**Jan 15**	0.62 (0.54, 0.70)
**Jan 16**	0.41 (0.35, 0.48)
**Jan 17**	0.27 (0.22, 0.32)
**Jan 18**	0.22 (0.17, 0.27)
**Jan 19**	0.18 (0.14, 0.23)
**Jan 20**	0.18 (0.13, 0.23)
**Jan 21**	0.15 (0.11, 0.20)
**Jan 22**	0.15 (0.10, 0.20)
**Jan 23**	0.13 (0.09, 0.18)
**Jan 24**	0.13 (0.08, 0.19)
**Jan 25**	0.13 (0.07, 0.19)
**Jan 26**	0.12 (0.06, 0.19)

**Jan 27**	0.10 (0.05, 0.18)
**Jan 28**	0.13 (0.06, 0.22)
**Jan 29**	0.14 (0.06, 0.25)
**Jan 30**	0.16 (0.06, 0.29)
**Jan 31**	0.17 (0.06, 0.33)
**Feb 1**	0.14 (0.04, 0.31)
**Feb 2**	0.18 (0.05, 0.39)
**Feb 3**	0.17 (0.03, 0.40)
**Feb 4**	0.07 (0, 0.26)
**Feb 5**	0.09 (0, 0.32)
**Feb 6**	0.11 (0, 0.40)

Table S4. Estimates of the effective reproduction number (R_t) in Suihua city

Date	Mean (95% Credible interval)
**Jan 16**	2.19 (1.94, 2.45)
**Jan 17**	1.99 (1.77, 2.21)
**Jan 18**	1.54 (1.37, 1.73)
**Jan 19**	1.31 (1.16, 1.47)
**Jan 20**	1.20 (1.06, 1.35)
**Jan 21**	0.84 (0.73, 0.96)
**Jan 22**	0.65 (0.55, 0.75)
**Jan 23**	0.53 (0.45, 0.62)
**Jan 24**	0.40 (0.33, 0.48)
**Jan 25**	0.41 (0.34, 0.49)
**Jan 26**	0.36 (0.29, 0.44)
**Jan 27**	0.27 (0.21, 0.35)
**Jan 28**	0.26 (0.20, 0.34)
**Jan 29**	0.21 (0.15, 0.28)
**Jan 30**	0.19 (0.12, 0.26)
**Jan 31**	0.18 (0.12, 0.26)
**Feb 1**	0.11 (0.06, 0.18)
**Feb 2**	0.11 (0.06, 0.19)
**Feb 3**	0.11 (0.05, 0.19)
**Feb 4**	0.07 (0.02, 0.15)
**Feb 5**	0.05 (0.01, 0.13)
**Feb 6**	0.02 (0, 0.08)
**Feb 7**	0.03 (0, 0.11)
**Feb 8**	0.04 (0, 0.14)

**Feb 9**	0.05 (0, 0.18)
**Feb 10**	0.06 (0, 0.23)
**Feb 11**	0.09 (0, 0.32)

Table S5. Estimates of the effective reproduction number (R_t) in Tonghua city

Date	Mean (95% Credible interval)
**Jan 18**	1.97 (1.59, 2.40)
**Jan 19**	1.54 (1.23, 1.87)
**Jan 20**	1.25 (1.00, 1.54)
**Jan 21**	1.12 (0.89, 1.38)
**Jan 22**	0.99 (0.79, 1.23)
**Jan 23**	0.59 (0.44, 0.77)
**Jan 24**	0.51 (0.37, 0.67)
**Jan 25**	0.50 (0.36, 0.66)
**Jan 26**	0.44 (0.31, 0.60)
**Jan 27**	0.34 (0.22, 0.48)
**Jan 28**	0.27 (0.16, 0.40)
**Jan 29**	0.20 (0.11, 0.32)
**Jan 30**	0.26 (0.14, 0.40)
**Jan 31**	0.17 (0.08, 0.31)
**Feb 1**	0.13 (0.05, 0.26)
**Feb 2**	0.11 (0.03, 0.23)
**Feb 3**	0.13 (0.03, 0.28)
**Feb 4**	0.11 (0.02, 0.27)
**Feb 5**	0.14 (0.03, 0.34)
**Feb 6**	0.06 (0, 0.21)
**Feb 7**	0.07 (0, 0.27)
**Feb 8**	0.09 (0, 0.34)

Table S6. Estimates of the effective reproduction number (R_t) in Haerbin city

Date	Mean (95% Credible interval)
**Jan 21**	3.34 (2.76, 3.97)
**Jan 22**	2.22 (1.81, 2.67)
**Jan 23**	1.91 (1.57, 2.29)
**Jan 24**	1.46 (1.18, 1.76)

**Jan 25**	1.25 (1.02, 1.51)
**Jan 26**	0.98 (0.78,1.20)
**Jan 27**	0.81 (0.63, 1.00)
**Jan 28**	0.67 (0.52, 0.85)
**Jan 29**	0.57 (0.43, 0.73)
**Jan 30**	0.39 (0.27, 0.52)
**Jan 31**	0.33 (0.22, 0.46)
**Feb 1**	0.19 (0.11, 0.29)
**Feb 2**	0.13 (0.06, 0.22)
**Feb 3**	0.06 (0.02, 0.12)
**Feb 4**	0.05 (0.01, 0.12)
**Feb 5**	0.02 (0, 0.07)
**Feb 6**	0.02 (0, 0.09)
**Feb 7**	0.03 (0, 0.11)
**Feb 8**	0.04 (0, 0.14)

Table S7. Estimates of the effective reproduction number (R_t) in Changchun city

Date	Mean (95% Credible interval)
**Jan 17**	3.15 (2.26, 4.18)
**Jan 18**	2.76 (2.00, 3.64)
**Jan 19**	1.73 (1.19, 2.38)
**Jan 20**	1.94 (1.40, 2.57)
**Jan 21**	1.85 (1.36, 2.42)
**Jan 22**	1.30 (0.91, 1.76)
**Jan 23**	0.99 (0.67, 1.38)
**Jan 24**	0.71 (0.45, 1.02)
**Jan 25**	0.67 (0.43, 0.98)
**Jan 26**	0.66 (0.42, 0.96)
**Jan 27**	0.32 (0.16, 0.54)
**Jan 28**	0.24 (0.10, 0.44)
**Jan 29**	0.23 (0.09, 0.42)
**Jan 30**	0.18 (0.06, 0.36)
**Jan 31**	0.16 (0.04, 0.35)
**Feb 1**	0.09 (0.01, 0.25)
**Feb 2**	0.11 (0.01, 0.30)
**Feb 3**	0.13 (0.02,0.36)
**Feb 4**	0.08 (0, 0.29)
**Feb 5**	0.10 (0, 0.37)
**Feb 6**	0.13 (0, 0.47)

Figure S1. Daily new COVID-19 infections and moving average curves in five worst-hit cities in China, Jan-Feb 2021. (A) Daily number of new infected individuals and the moving average processed curve (n = 7) in Shijiazhuang city. (B), (C), (D) and (E). The same as A but in Suihua, Tonghua, Haerbin and Changchun cities respectively.

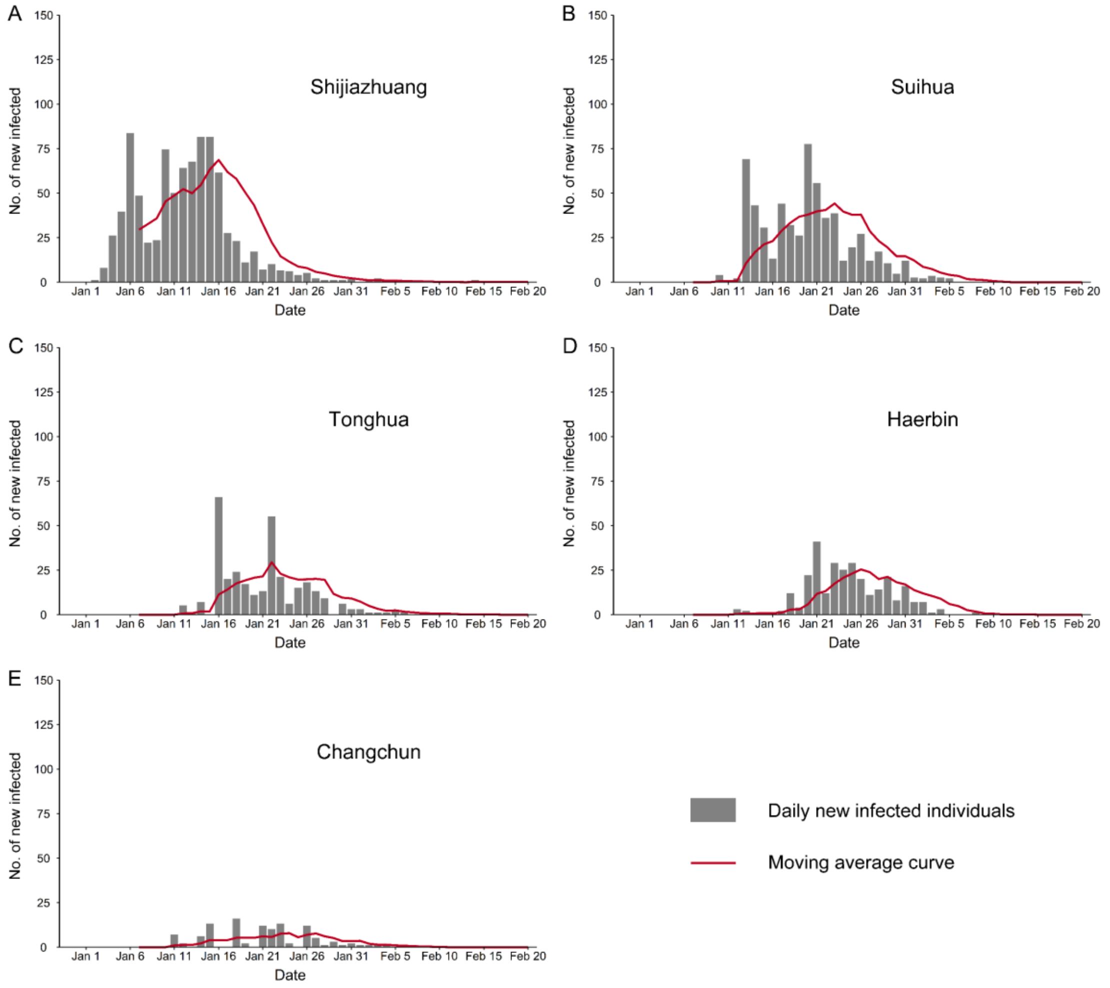

Figure S2. Estimates of R_0 in five worst-hit cities with different generation time distributions. R_0 = basic reproduction number.

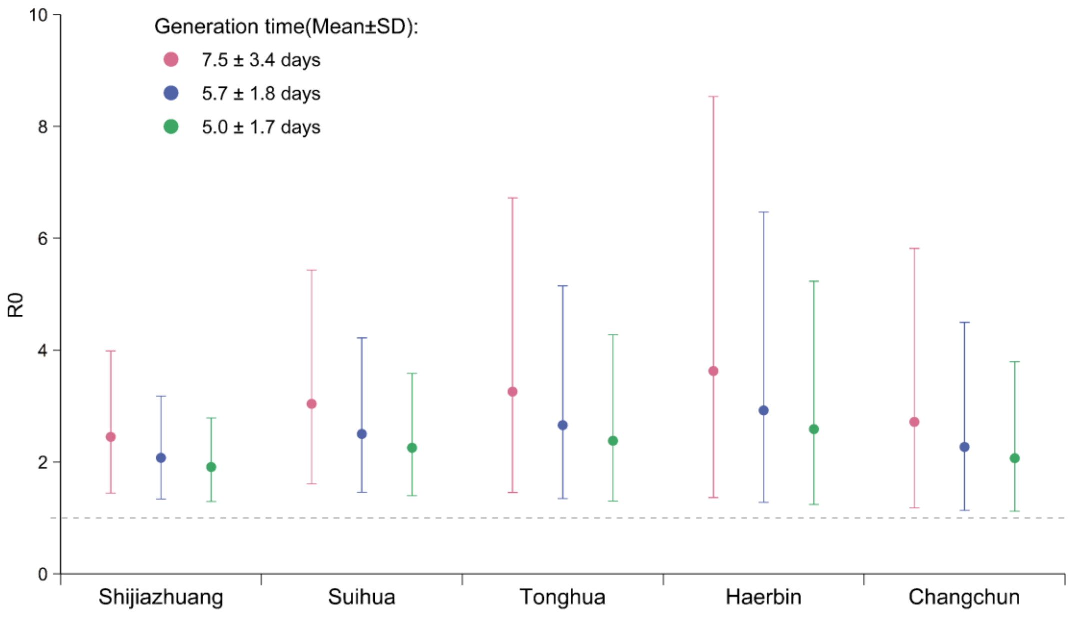

Supplementary References

1. The State Statistical Bureau. The Bulletin of the Seventh National Census. 2021.
2. Cori A, Ferguson NM, Fraser C, Cauchemez S. A new framework and software to estimate time-varying reproduction numbers during epidemics. American journal of epidemiology. 2013;178(9):1505-12.
3. Thompson RN, Stockwin JE, van Gaalen RD, Polonsky JA, Kamvar ZN, Demarsh PA, et al. Improved inference of time-varying reproduction numbers during infectious disease outbreaks. Epidemics. 2019;29:100356.
